# Pain in Child Health from 2002 to 2015: The early years of an international research training initiative

**DOI:** 10.1080/24740527.2018.1562844

**Published:** 2019-02-22

**Authors:** Carl L. von Baeyer, Bonnie J. Stevens, Kenneth D. Craig, G. Allen Finley, C. Celeste Johnston, Ruth V.E. Grunau, Christine T. Chambers, Rebecca R. Pillai Riddell, Jennifer N. Stinson, Patrick J. McGrath

**Affiliations:** a University of Saskatchewan, Saskatoon, SK, Canada; b University of Toronto, Toronto, ON, Canada; c University of British Columbia, Vancouver, BC, Canada; d Dalhousie University, Halifax, NS, Canada; e McGill University, Montreal, QC, Canada; f York University, Toronto, ON, Canada

**Keywords:** research methods, education, children, pediatric pain, developmental

## Abstract

**Background**: The 2018 Global Year for Excellence in Pain Education, an initiative of the International Association for the Study of Pain, brought worldwide attention to the need for education that crosses narrow disciplinary boundaries, addresses up-to-date research methods and findings, and encourages teamwork among trainees and mentors at different levels of training and with different perspectives.

**Aims**: This commentary describes the development of Pain in Child Health (PICH), an interdisciplinary training program for researchers in pediatric pain at the undergraduate, graduate, and postdoctoral levels of training.

**Methods**: Based on documentation of the structure, training processes, leadership, and membership of PICH, we outline its organization and its challenges and accomplishments over the first 12 years of its growth into a well-known international program.

**Results and Conclusions**: Pain in Child Health began as a Strategic Training Initiative of the Canadian Institutes of Health Research in 2002 and developed into an international research training consortium featuring cross-site and cross-discipline mentorship and collaboration. PICH trainees and alumni have contributed extensively to the current scientific literature on children’s pain. PICH could serve as a possible model for training and mentorship in other specialized health research domains within and outside thestudy of pain.

## RÉSUMÉ

**Contexte**: En 2018, l’Année internationale pour l’excellence en éducation sur la douleur, une initiative de l’Association internationale pour l’étude de la douleur, a attiré l’attention partout dans le monde sur l’importance d’une éducation qui transcende les frontières étroites entre les disciplines, qui aborde les méthodes et les résultats de la recherche les plus actuels et qui encourage le travail d’équipe parmi les apprenants et les mentors à différents niveaux de formation et à partir de différentes perspectives.

**But**: Ce commentaire décrit l’évolution de *Pain in Child Heath* (PICH), un programme de formation interdisciplinaire destiné aux chercheurs en douleur pédiatrique à tous les niveaux de formation : premier cycle, cycles supérieurs et postdoctoral.

**Méthodes**: À partir de la documentation portant sur la structure, les processus de formation, le leadership et les caractéristiques des membres du PICH, nous décrivons son organisation, ses difficultés et ses accomplissements au cours de ses premiers douze ans, jusqu’à ce qu’il devienne un programme international de renom.

**Résultats et conclusions**: À ses débuts, *Pain in Child Health* était une initiative de formation stratégique des Instituts de recherche en santé du Canada en 2002. Il s’est par la suite transformé en consortium international de formation à la recherche axé sur le mentorat et la collaboration entre différents sites et différentes disciplines. Les étudiants et les anciens du PICH ont largement contribué à la littérature scientifique sur la douleur pédiatrique. Le PICH pourrait possiblement servir de modèle en matière de formation et de mentorat dans d’autres domaines de recherche spécialisée en santé, que ce soit dans le contexte de l’étude de la douleur ou dans un autre contexte.

The 2018 Global Year for Excellence in Pain Education, an initiative of the International Association for the Study of Pain, brought worldwide attention to the need for increased education in pain, particularly education that crosses narrow disciplinary boundaries, that addresses up-to-date research methods and findings, and that encourages teamwork among trainees and mentors at different levels of training and with different perspectives.^1^

Grounded in this perspective, our commentary describes Pain in Child Health (PICH), an interdisciplinary training program for researchers in pediatric pain at the undergraduate, graduate, and postdoctoral levels of training. We outline its inception and organization and the first 12 years of its development into a well-known international program. It could serve as a possible model for training and mentorship in other specialized health research domains within and outside the study of pain.

## Inception

PICH was conceived on a Saturday morning in early 2001 at the Farmers Market in Halifax, by Patrick McGrath and Allen Finley, at one of their weekly visits for coffee. They had seen a call for strategic research training initiatives from the Canadian Institutes of Health Research (CIHR) and wondered what they could do in the field of pediatric pain. At the time, pain researchers focusing on childhood often felt marginalized within their departments, across all disciplines. Research on adults’ pain was much more visible.

Based on a preliminary proposal, CIHR provided a small grant to get a group of coinvestigators together. McGrath and Finley invited researchers from across Canada who specialized in pediatric pain and had advanced research trainees. The founding group of co-principal investigators (co-PIs), and the PICH Management Committee from 2002 through 2009, were as follows (Table 1).

**Table 1.** Co-principal investigators for first PICH grant, 2002–2009.

Patrick McGrath	Psychology	Dalhousie University and IWK Health Centre
Allen Finley	Anaesthesia	Dalhousie University and IWK Health Centre
Kenneth Craig	Psychology	University of British Columbia
Bonnie Stevens	Nursing	University of Toronto and Hospital for Sick Children
Carl von Baeyer	Psychology	University of Saskatchewan and Royal University Hospital
Celeste Johnston	Nursing	McGill University and Montreal Children’s Hospital

PICH = Pain in Child Health.

They met in Halifax for a grant writing weekend. The application, following much polishing, was submitted and was successful, supporting the founding of “Pain in Child Health—A Strategic Training Initiative of the Canadian Institutes of Health Research” with a 6-year grant of nearly $2 million to run from 2002 through 2008.

At an early meeting of the co-PIs in Winnipeg in 2002, an ambitious program of activities was laid out (Table 2). Most of these programs were offered starting in the first years of PICH, and additional programs including extensive international collaboration were added to this preliminary list.

**Table 2.** May 2002 brainstorm list of activities planned for PICH, with notes on later implementation.

Concept or plan (2002)	Notes on later implementation (2018)
Annual, week-long summer/fall institute with a concentrated curriculum on research methods	Training institutes (workshops) were 1–3 days and usually associated with another conference; see Table 4
Biweekly, national research teleconference with data display capabilities	These became monthly international webinars
Sophisticated electronic communication system across the research centres	Email listserv and resources shared on website
Web-based education modules/courses for trainees	One course was developed for PICH, on measurement and assessment of pain
Visits by trainees and faculty to other research centers to learn techniques, develop new methodologies, and collaborate on grant applications and research in progress	As planned
Transdisciplinary and cross-center supervision of research trainees	As planned
Mentoring workshop for faculty and trainees	Held periodically at training institutes
Visiting speakers program of distinguished scientists	As planned but rare
Partnerships with industry (pharmaceutical, communications), government (Health Canada, Provincial Departments of Health), universities, health centers, pediatric research foundations, other training consortia, funding agencies (CIHR, SSHRC, NSERC, CHSRF, provincial agencies), professional associations, and pain-related organizations, (CUREPAIN2, Canadian Pain Society)	As planned but limited; see Figure 2
Evaluation of the structure, process, and outcome of our collaborative research program	Completed for PICH renewal, 2009
Dissemination program for pediatric pain research to clinicians, policymakers, and the public	Extensive publications and international conference presentations identified as PICH products. Public engagement (e.g., with parents) increased in 2015 with PICH2GO

PICH = Pain in Child Health; CIHR = Canadian Institutes of Health Research; SSHRC = Social Sciences and Humanities Research Council of Canada; NSERC = Natural Sciences and Engineering Research Council of Canada; CHSRF = Canadian Health Services Research Foundation.

**Figure 1.**
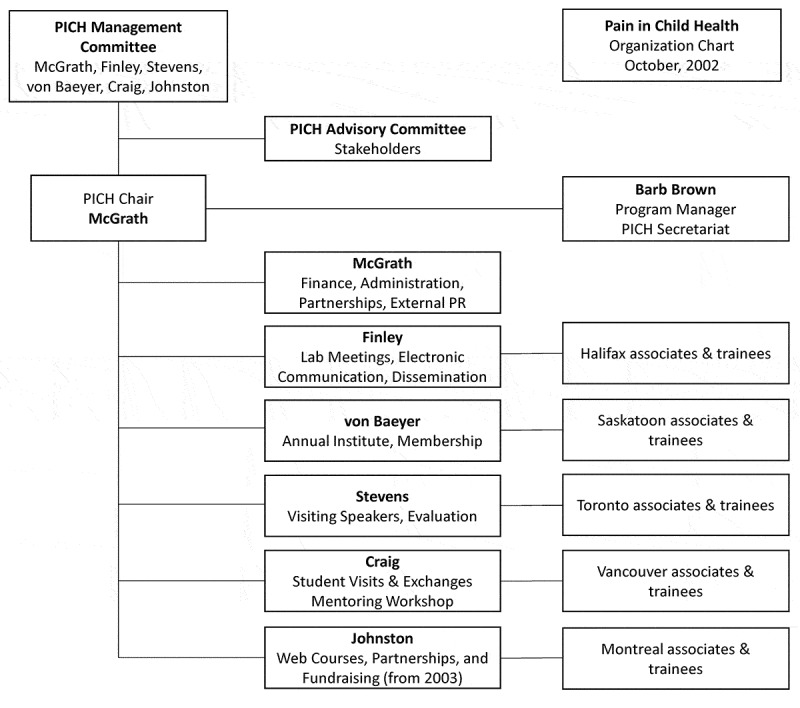
Organizational chart for PICH as of October 2002. Faculty mentioned as “associates” were co-investigators on the CIHR grant.

The administrative structure of the program as of 2002 is shown as an organizational chart in Figure 1.

Additions were later made to the group of co-PIs as shown in Table 3.

**Table 3.** Additional co-principal investigators for second CIHR grant, 2009–2015 (extended to 2018).

2009 (As part of the successful application for a 6-year renewal of the CIHR grant)		
Ruth Grunau	Psychology	University of British Columbia and BC Child and Family Research Institute
2013 (as part of a succession plan for PICH leadership)		
Jennifer Stinson	Nursing	University of Toronto and Hospital for Sick Children
Christine Chambers	Psychology	Dalhousie University and IWK Health Centre
Rebecca Pillai Riddell	Psychology	York University and Hospital for Sick Children

CIHR = Canadian Institutes of Health Research; PICH = Pain in Child Health.

In 2015, Bonnie Stevens took over from Patrick McGrath as nominated principal investigator and chair of the PICH Management Committee, and the headquarters of PICH was moved to the SickKids Centre for Pain Management, Research and Education (Pain Centre) in Toronto.

**Figure 2.**
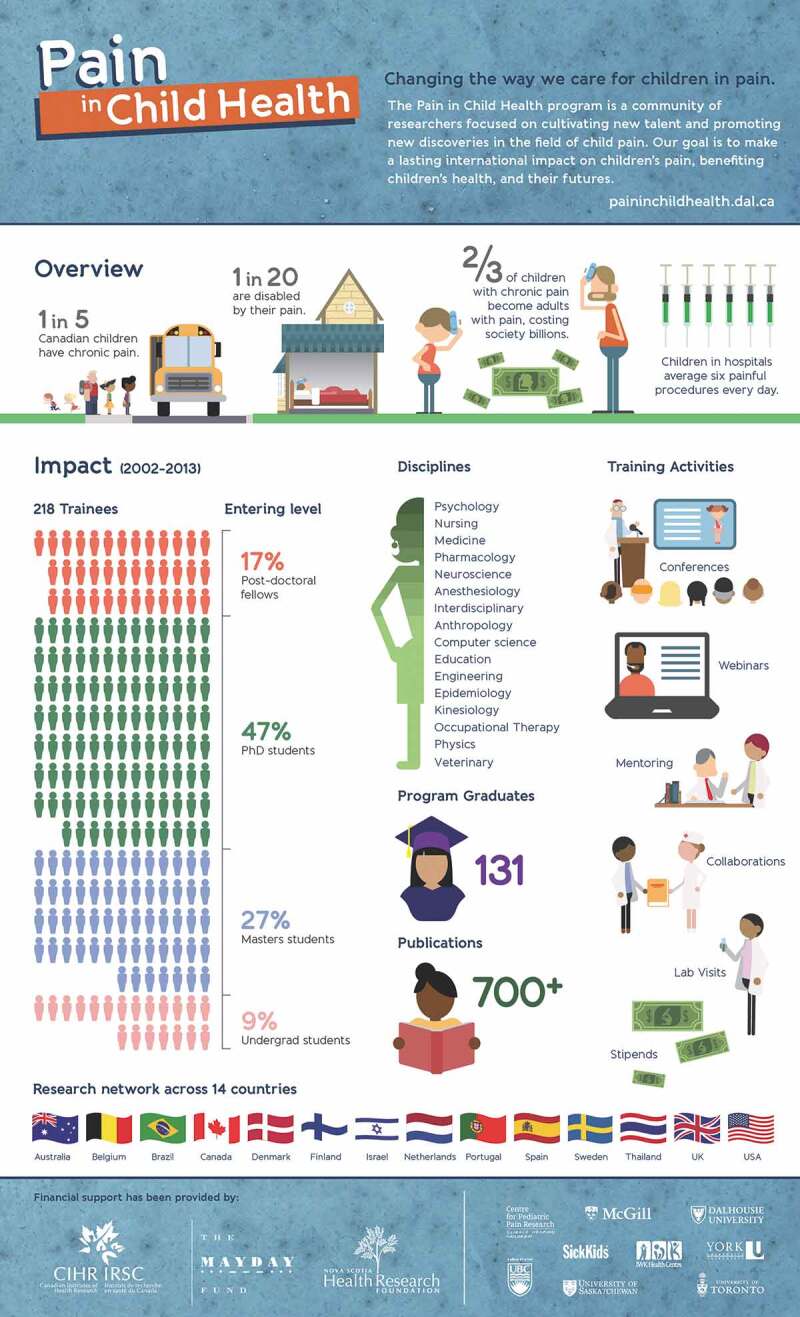
Infographic from 2014 summarizing PICH activities in its first 12 years. The 2018 version, adapted for the new location and funding sources at SickKids, is at www.sickkids.ca/PICH/key-info

The two major grants from CIHR (2002–2009 and 2009–2018) provided funds for Canadian PICH trainees’ stipends and travel to training institutes and lab visits as well as for PICH administration to support training activities. The participating universities contributed to funding of their own trainees’ PICH activities. In addition, an unrestricted educational grant was received from Janssen-Ortho Pharmaceuticals, and funding was also received from the Nova Scotia Health Research Foundation. The Mayday Fund, a private charitable foundation based in New York and dedicated to the alleviation of pain, provided generous financial support for trainees who were not based at Canadian universities. With the invaluable support of the Mayday Fund and its Executive Director Christina Spellman, by 2013 trainees and faculty from 14 countries were participating in PICH: see the infographic in Figure 2.

## Trainees

Starting immediately with the award of the first CIHR grant in 2002, trainees were recruited with their supervisors’ support. Most trainees came from the disciplines of psychology and nursing, with fewer from other health care disciplines such as medicine, pharmacy, physical therapy, and neuroscience. The first groups of trainees in 2002 were Canadian; from 2003 onward international trainees were accepted. All of the first intake of international trainees (2003–2005; *n* = 11) and almost all of the Canadian trainees remained active in pediatric pain work after their PICH years. By 2009, over 200 trainees had joined PICH, and by 2018, over 300 had joined.

## Activities and impact

Though trainees and mentors met periodically at conferences and in monthly webinars, the core of PICH activities was training workshops (institutes), generally around 2 days in length, which were held once or twice a year across Canada. From 2002 through 2015, 18 such workshops were held. A list of the training institutes with their locations and topics is shown in Table 4. Every second year these were held in conjunction with the International Forum on Pediatric Pain in Nova Scotia. Other workshops were held in conjunction with national and international pain conferences, including the World Congress on Pain and the International Symposium on Pediatric Pain, to leverage the benefits of trainee travel. All trainees attending these institutes were expected and supported to stay for the conference and strongly encouraged to present posters. In 2014, a workshop emphasizing neuroscience was held with another CIHR Strategic Initiative on Health Research, Molecules to Community (PainM2C). PICH activities are further documented in *PICH Pulse*, a newsletter that has been published regularly from 2002 through the present (www.sickkids.ca/PICH/key-info/PICH-pulse)

**Table 4.** PICH training workshops (Institutes) from 2002 through 2015.

No.	Institute date	Location	Main theme	Attendance (Canadian/international trainees)
18th	September 29–October 1, 2015	White Point, Nova Scotia	Tools for Your Career as a Pediatric Pain Researcher	25/19
17th	May 23–25, 2014	Québec CIty, Québec	Leaders in Neuroscience of Chronic Pain: Going from Mission Impossible to Mission Possible	24/16
16th	October 11–13, 2013	White Point, Nova Scotia	Pediatric Pain Pharmacology	27/18
15th	January 3–6, 2013	Winnipeg, Manitoba	RCT Boot Camp: Design, Implementation and Interpretation of Randomized Controlled Trials	29/13
14th	May 21–23, 2012	Whistler, British Columbia	Career Development. Translating Neuro-Developmental Research Into Clinical Application	29/11
13th	October 11–13, 2011	White Point, Nova Scotia	The Future of Research for Pain in Children	30/14
12th	August 26–29, 2010	Montréal, Québec	What’s in Your Toolbox? Methods for Pediatric Pain Research	23/15
11th	November 15–17, 2009	Toronto, Ontario	Research Ethics	23/10
10th	September 30–October 2, 2008	White Point, Nova Scotia	Media Training	23/10
9th	May 24–27, 2008	Victoria, British Columbia	Policy Research	26/10
8th	May 14–17, 2007	Val-Morin, Québec	Knowledge Transfer	25/10
7th	October 11–12, 2006	White Point, Nova Scotia	Walking the Tightrope: Balancing Success in Work & Home Life	21/5
6th	June 21–24, 2006	Vancouver, British Columbia	Long-Term Effects of Pain and Chronic Pain	29/8
5th	May 8–11, 2005	Oak Island, Nova Scotia	Mentoring and Ethics	25/4
4th	October 13–14, 2004	White Point, Nova Scotia	Knowledge Dissemination	24/5
3rd	May 2–5, 2004	Harrison Hot Springs, British Columbia	Early Research Career Skill Development	25/4
2nd	May 19–21, 2003	Toronto, Ontario	Did It Work? Evaluating Pain-Relieving Interventions	16/1
1st	September 17–19, 2002	White Point, Nova Scotia	What’s Special About Children?	19/0

PICH = Pain in Child Health.

A previous study of the impact of PICH used quantitative and qualitative methods to document how it has helped to create a global community of researchers in pediatric pain up to 2013.^2^ Many international collaborations have been fostered through lab visits and conferences. The impact of PICH is seen partly in trainees’ publications: by 2014, over 700 unique articles had been published by PICH trainees.^2^ Publications led by PICH trainees have appeared in journals of pain, pediatrics, anesthesiology and other medical disciplines, nursing, neuroscience, psychology, physiotherapy, computer science, and medical anthropology, published in North and South America, Europe, Australia, and Asia. Moreover, according to a bibliometric study, nearly 9% of all identified articles on pediatric pain published between 2003 and 2010 were by a first or senior author affiliated with PICH.^3^

In 2012, under the direction of the Canadian minister of industry, the Council of Canadian Academies released a report entitled *The State of Science and Technology in Canada*.^4^ Pediatric pain was identified as first in research productivity in a list of the top 10 Canadian highly specialized research clusters. Canada’s share of world publications on pediatric pain, at 15.5%, was greater than Canada’s proportionate share of world research on the environment, fisheries, geology, oil, gold, and other major Canadian investigative themes. It is likely that the success of PICH over the previous decade contributed to this accomplishment.

## Present and future opportunities

The strong foundational years of PICH have resulted in a global network of interdisciplinary pediatric pain researchers that has contributed significantly to enhancing research capacity. The current Canadian leaders of PICH are broadening this network to include scholars from other countries and clinical disciplines, as well as basic and knowledge translation scientists, patients, clinicians, and educators. PICH has disseminated new knowledge of pediatric pain broadly and is continuing to grow with the help of knowledge translation strategies newly being adopted in science, including social media. Online pediatric pain curricula and networking opportunities in local and regional meetings in different continents bolster the existing monthly PICH webinars, lab exchanges, and mentoring opportunities.

As well as securing funding, which required continuous effort, PICH leaders have worked hard to engage trainees and faculty from disciplines outside of psychology and nursing. Many other disciplines such as medicine, pharmacology, neurobiology, kinesiology, education, medical anthropology, computer science, and physical and occupational therapy were represented by only a few participants. This represents an opportunity for growth of the PICH community in Canada and worldwide.

PICH continues to bring together trainees and researchers in Canada and many other countries. With the initial co-PIs having transitioned into advisory roles, many lead roles are occupied by a new generation of PICH scholars. They actively seek external partnerships to support foundational activities such as the monthly webinars as well as new formats for meetings and other new activities that innovatively strengthen PICH impacts across the spectrum of stakeholders. PICH members have access to many other resources through the SickKids Pain Centre. Current developments include a web-based, free, internationally accessible set of 10 online training modules (Online Pediatric Pain Curriculum) and a new model for training workshops (PICH2GO). These new developments include outreach to patients and families and greater involvement of clinicians. Current and future plans are shown on the PICH website, www.sickkids.ca/PICH.
